# Supplementary material for: Pooled prevalence and associated factors of diarrhea among under-five years children in East Africa: A multilevel logistic regression analysis
Source: PLoS One. 2022 Apr 14;17(4):e0264559. doi: 10.1371/journal.pone.0264559 (PMC9009646; doi:10.1371/journal.pone.0264559)
Supplement: Attachment — Submitted filename: comment.docx [file pone.0264559.s002.docx]

**Comment**

**General comment**

- The whole manuscript should be revised by English language expert as it is full of grammatical errors
- What will add this paper from the previous similar studies at sub Sahran African level (e.g: *Demissie GD, Yeshaw Y, Aleminew W, Akalu Y (2021) Diarrhea and associated factors among under five children in sub-Saharan Africa: Evidence from demographic and health surveys of 34 sub-Saharan countries. PLoS ONE 16(9): e0257522. https://doi.org/10.1371/journal. pone. 0257522 )*. At least you did not recognize by using these studies as reference. You have to also identify the major gap of these studies that you filled.
- Implication and justification for the significantly associated variables the discussion part is not strong and hence should be revised

**Background**

- Sentence on Line 51 and 52 should be clear. “Global 51 burden of diseases 2019 report shows that sub-Saharan total DALYs estimate due to diarrhea was 52 13.01% respectively (3, 4).”
- Line 57 and 58: Reference should be incorporated

**Methods and materials**

- The sample size should be weighted sample size and you did not mention if it is weighted otherwise it should be
- You have to also prepare a table which explains that survey years and weighted sample size for each country
- Line number 98 and 99 is repeated
- Time to fetch drinking water source should be included as one variable as it it is already in DHS data sets
- Why did you include “community level of poverty and community level of illiteracy variables” as community level factors as these variables are already included in the level 1 as household wealth index and education status as individual level?
- From your operational definition(media exposure), I hope it is a composite variable and you have to explain this
- Category of your dependent and independent variables should be supported by references
- Part of data analysis is very long paragraphs. Please divide each in to more than two parts(line number 144-166)

**Result**

- You have to revise measurement of a variable(distance to health facility)
- Line 249 “multivariate” is not correct word as your outcome variable is binary. So use the word “multivariable”
- Line number 259 spelling error

**Discussion**

- Line number 300-306 is part of result part. Please avoid this from discussion part
- You did not study “incidence” so avoid it Line number 308
- Justifications should be supported by evidences(references) in the discussion part
- I did not see your significant community level variables in your discussion

**References**

- Revise your References citation based on the standard
