# Supplementary material for: Pooled prevalence and associated factors of diarrhea among under-five years children in East Africa: A multilevel logistic regression analysis
Source: PLoS One. 2022 Apr 14;17(4):e0264559. doi: 10.1371/journal.pone.0264559 (PMC9009646; doi:10.1371/journal.pone.0264559)
Supplement: Attachment — Submitted filename: Response to Reviewers.docx [file pone.0264559.s005.docx]

**Author’s response to reviews**

**Title: Pooled prevalence and associated factors of diarrhea among under-five years children in East Africa: A multilevel logistic regression analysis**

Authors:

Abiyu Abadi Tareke (abiyu20010@gmail.com)

Ermias Bekele Enyew ([Ermiashi@gmail.com](mailto:Ermiashi@gmail.com))

Bayley Adane Takele (behaileadane@gmail.com)

Version: 2

Date: 3 February 2022

**Author’s response to reviews:**

**Plos one**

**Point by point response for editors/reviewers comments**

**Manuscript title:** Pooled prevalence and associated factors of diarrhea among under-five years children in East Africa: A multilevel logistic regression analysis

**Manuscript ID:** PONE-D-21-28429

**Dear editors/reviewers:**

Dear all,

We would like to thank you for your generous, revealing and constructive comments to the second version of the manuscript. You valuable comments would advance the substance and content of the manuscript. The authors accounted each comments and clarification questions of editors and reviewers in a focused way.

Our point-by-point responses to each comment and questions are detailed on the following pages. Further, the details of changes were shown by track changes in the supplementary document attached.

**Comments**

- Point by point responses with LINE number of corrected sentences would facilitate to review
- Attaching the old version of manuscript makes me confused
- **Author’s response: We made correction to this comments**
- English language correction is deemed necessary.
- **Author’s response: We made some correction to this comment**
- Introduction should be clear, concise, and identify the added scientific value from the study.
- **Author’s response: Some scientific values are added**
- Table 2 – What is the difference between variables ‘mother’s current working status’ and ‘mother’s working status’. The proportion are different.
- **Author’s response: Thank you reviewer for your constructive comment. We accept and modified it. The variable named “mother’s current working status” was added to the table mistakenly. The difference in the proportion is occurred because of this variable was tabulated without adding the command of sample weighting. That way it has lower frequency compared to the second variable ‘mother’s working status’. As the variable ‘mother’s working status’ has weighted sample we decided to continue with this variable and we removed the variable having un-weighted frequency.**
- You cannot control both ‘maternal education status’ and ‘community level educational status’ together in a regression model. According to your methodology, these two are derived from a single variable/information, correct? Same applied to poverty/wealth index.
- **Author’s response: we thank you for your valuable comment. Community level factors are pertinent factors for children health services, we considered the community level factors by aggregating them from the respective individual level factors to indicate the neighboring effect. This helps policymakers to take intervention at both individual and community levels. For example, child born from mother who is from communities with lower level of community education might be clustered in specific location and taking appropriate intervention in this group of women could have a great advantage to increase child health services including diarrhea prevention and treatment services. In our opinion educational status at individual level are different from educational status generated at community level.**
- Also, did you check collinearity?
- **Author’s response: Thank you reviewer for your concern. Yes, we have checked it. The multicollinearity issue was addressed using pseudo linear regression analysis through applying the command “estat vif” and the mean variance inflation factor (VIF) of 2.31. Which ensure that non –presence of multicollinearity b/n predictor variables.**
- Regarding relation between diarrhea and time of breast feeding initiation, I also do not think these are related. That’s the reason why I am asking to you why because you study population is under 5 children and you controlled breastfeeding initiation in the regression model.
- **Author’s response: Time of breastfeeding initiation is included in this study because of early initiation of breast feeding has many health benefit. According to WHO feeding colostrum in the first hour increases the likelihood babies will continue to be breastfed which gives them a head start in the “race against** [**malnutrition**](https://www.medicalnewstoday.com/articles/179316)**“.  This means children who had history of early breast feeding initiation are less likely to encounter malnutrition and if the probability of encountering malnutrition is low the probability of diarrhea will also decrease. Additionally, the more initiation of breast feeding early the more likely to continue their breast feeding properly. And this will reduce the frequency of diarrhea. Therefore, we included this variable considering those scientific backgrounds.**
- Discussion section needs to be improved a lot.
- **Author’s response: Thank you a lot for your comment. We accept all your comments and modified it accordingly.**
