# Supplementary material for: Pooled prevalence and associated factors of diarrhea among under-five years children in East Africa: A multilevel logistic regression analysis
Source: PLoS One. 2022 Apr 14;17(4):e0264559. doi: 10.1371/journal.pone.0264559 (PMC9009646; doi:10.1371/journal.pone.0264559)
Supplement: Attachment — Submitted filename: Reviewer comments.docx [file pone.0264559.s001.docx]

**PONE-D-21-28429**

**Pooled prevalence and associated factors of diarrhea among under-five years children in East Africa: A multilevel logistic regression analysis**

This manuscript describes the pooled prevalence of diarrhea among East Africa countries and its predictors using the DHS data. While the manuscript is of some interest, the manuscript could be strengthened by several modest changes as outlined below.

Comments

- English language needs to be improved and edited by a native English speaker.
- East Africa is repeated in keywords.
- LINE 47 – Do you mean “second most common cause of death”?
- A brief introduction of East Africa (e.g. rural/urban ratio, characteristics related to diarrhea and current WASH program in East Africa in the Methods section would be helpful to the readers.
- Literacy and education are different and could not be able to categorize as literacy according to educational level.
- If you control both maternal education and education of caregiver (considered mostly would be mothers), there is an collinearity issue.
- It is better to rearrange the categories among variables in order in Table. (e.g. 15-24 yrs first followed by 25-34, 35-49, same apply to education, wealth index, etc..)
- Some variables mentioned in the methods such as media exposure are missing in Table 1.
- Clear explanation of recoding the variables is deemed necessary. I noticed that you explained for some (but still need better explanation) while missing for some variables (e.g. distance from health facility)
- LINE 176, 177 – what do you mean by the proportion in the 95%CI. Mean should come with SD while median should come with IQR.
- Any relation between diarrhea and time of breastfeeding initiation for children > 2 years?
- Why do you include Visit to health facility or visited by health worker 🡪 visit to health facility due to diarrhea? If yes, which happened after diarrhea, should not be controlled for it. Otherwise, please explain.
- For pooled prevalence in the forest plot, are there any common characteristics between countries (e.g. low income countries, lower middle income countries or high income countries)? If yes, you can try a sub-group analysis because there is a big difference in prevalence among countries. You can also explore the heterogeneity by the I-square value.
- Adjusting the scale in the x-axis of forest plot will give a better picture of point estimate and 95%CI.
- I appreciated that the authors tried to do sensitivity analysis here. As Table 2 and Figure 2 provide same information, you can omit one.
- Double check on the sensitivity analysis result. As you mentioned, although Ethiopia has a great influence on the pooled prevalence, in fact, Madagascar has lowest prevalence and weight % is not so much different from Ethiopia but result does not change in sensitivity analysis while prevalence becomes doubled after omitting Ethiopia.
- Figure 2 is not eye catching and the number are overlapped. Is the x-axis percentage? Better redraw it.
- LINE 218-236 – remove the subtitles and explanation of Table 2 results should go under the subtitle ‘Model comparison’.
- It is interesting that type of toilet is not associated with diarrhea. Is there any open defecation culture and such kinds of variables included in the dataset? If yes, you should consider it. Are every household have a toilet?
- As Figure 2 and Table 3 give same information, omit one.
- Title for Table 3 is missing.
- LINE 249-259 – can omit as there is no information there.
- The authors tend to cover all information in the text and again in the table. You should only include the most important details in the text when you also have a table.
- The discussion is weak in light of the findings. The discussion section should be rewritten. The ideas are incoherently mixed. The discussion needs to focus on the key implications of the data with a separate paragraph for each concept.
- There are repetition of results in the discussion.
- First paragraph of discussion, should start with brief answer to your research question. Do you think the diarrhea prevalence in East Africa is low or high? And why do you think? Is there any target? Etc.
- In my perspective, comparing the pooled prevalence of East Africa and the prevalence of e.g. Ethiopia is not meaningful because Ethiopia is already included in the pooled prevalence. Furthermore, you mentioned the reason as ‘dissimilarity’ between East Africa and e.g. Ethiopia also not meaningful.
- LINE 316-317 is confusing. Children have to provide supplementary food when the time arrives and it cannot be concluded as ‘highly likely to ingest unhygienic foods’. It mainly depends on the caregivers’ knowledge.
- The defecation culture in East Africa (?open defecation), any efforts in WASH program, government’s implementation, caregiver’s knowledge, etc. should be discussed.
