# Supplementary material for: Pooled prevalence and associated factors of diarrhea among under-five years children in East Africa: A multilevel logistic regression analysis
Source: PLoS One. 2022 Apr 14;17(4):e0264559. doi: 10.1371/journal.pone.0264559 (PMC9009646; doi:10.1371/journal.pone.0264559)
Supplement: Attachment — Submitted filename: Reviewer comments.docx [file pone.0264559.s004.docx]

**PONE-D-21-28429R1**

**Pooled prevalence and associated factors of diarrhea among under-five years children in East Africa: A multilevel logistic regression analysis**

Thank you for the revised version. Although there is some improvement, the manuscript still requires a significant improvement from the previous version of the manuscript. My sense is that the authors could not sufficiently explain or solve the reviewer’s comments and its current form does not have sufficient quality to warrant publication in PLOS ONE.

Comments

- Point by point responses with LINE number of corrected sentences would facilitate to review
- Attaching the old version of manuscript makes me confused
- English language correction is deemed necessary.
- Introduction should be clear, concise, and identify the added scientific value from the study.
- Table 2 – What is the difference between variables ‘mother’s current working status’ and ‘mother’s working status’. The proportion are different.
- You cannot control both ‘maternal education status’ and ‘community level educational status’ together in a regression model. According to your methodology, these two are derived from a single variable/information, correct? Same applied to poverty/wealth index.
- Also, did you check collinearity?
- Regarding relation between diarrhea and time of breast feeding initiation, I also do not think these are related. That’s the reason why I am asking to you why because you study population is under 5 children and you controlled breastfeeding initiation in the regression model.
- Discussion section needs to be improved a lot.
